# Improving Sprint Performance in Soccer: Effectiveness of Jump Squat and Olympic Push Press Exercises

**DOI:** 10.1371/journal.pone.0153958

**Published:** 2016-04-21

**Authors:** Irineu Loturco, Lucas Adriano Pereira, Ronaldo Kobal, Thiago Maldonado, Alessandro Fromer Piazzi, Altamiro Bottino, Katia Kitamura, Cesar Cavinato Cal Abad, Miguel de Arruda, Fabio Yuzo Nakamura

**Affiliations:** 1 NAR–Nucleus of High Performance in Sport, São Paulo, SP, Brazil; 2 Palmeiras Sport Society, São Paulo, SP, Brazil; 3 University of Campinas, Campinas, SP, Brazil; 4 State University of Londrina, Londrina, PR, Brazil; West Virginia University School of Medicine, UNITED STATES

## Abstract

Training at the optimum power load (OPL) is an effective way to improve neuromuscular abilities of highly trained athletes. The purpose of this study was to test the effects of training using the jump squat (JS) or Olympic push-press (OPP) exercises at the OPL during a short-term preseason on speed-power related abilities in high-level under-20 soccer players. The players were divided into two training groups: JS group (JSG) and OPP group (OPPG). Both groups undertook 12 power-oriented sessions, using solely JS or OPP exercises. Pre- and post-6 weeks of training, athletes performed squat jump (SJ), countermovement jump (CMJ), sprinting speed (5, 10, 20 and 30 m), change of direction (COD) and speed tests. To calculate the transfer effect coefficient (TEC) between JS and MPP OPP and the speed in 5, 10, 20, and 30 m, the ratio between the result gain (effect size [ES]) in the untrained exercise and result gain in the trained exercise was calculated. Magnitude based inference and ES were used to test the meaningful effects. The TEC between JS and VEL 5, 10, 20, and 30 m ranged from 0.77 to 1.29, while the only TEC which could be calculated between OPP and VEL 5 was rather low (0.2). In addition, the training effects of JS on jumping and speed related abilities were superior (ES ranging from small to large) to those caused by OPP (trivial ES). To conclude, the JS exercise is superior to the OPP for improving speed-power abilities in elite young soccer players.

## Introduction

The evolution of top-level soccer technical and physical performance demands the progressive development of neuromuscular abilities related to engagement in match-related powerful activities [1], such as sprinting and jumping. Improving these abilities is a challenge in soccer, especially during periods of high exposure to endurance training (i.e., preseason), whereby the interference effect might impair power and speed performance adaptations [2–4]. Nevertheless, training at the “optimum power loads (OPL)” (i.e., the load capable of maximizing the muscle power output measured in the bar) in half-squat and jump squat (JS) exercises was effective in counteracting the power and speed decrements that commonly occur in elite soccer players during the short preseasons [2].

In addition, it was reported that training at the optimum power zone elicited similar neuromuscular improvements to those observed after traditional strength training [5]. Importantly, throughout the in-season period (during which training volume is reduced compared with the preseason), the classic strength-power periodization (as proposed by Plisk and Stone [6]) proved to be less effective at improving speed-power related abilities in elite soccer players than training “solely” at the optimum power zone using the JS exercise (unpublished results). Although this training mode (i.e., JS at the OPL) seems to be an effective and practical alternative for enhancing the neuromechanical performance of soccer players during the in-season period, it remains to be established whether the JS “per se” is the most appropriate exercise to be used in the training programs of elite athletes. In addition, as both exercises are “vertically oriented” and this training axis showed to be superior to “horizontally oriented” exercises to enhance 20-m speed performance in elite soccer players (who performed unloaded jumps) [7], it would be valuable to test their effectiveness under loaded conditions (i.e., JS and OPP at the OPL).

Strength and conditioning coaches habitually use complex multi-joint strength-power exercises as specific strategies to improve the neuromuscular performance of top-level athletes. In this regard, Olympic weight lifts are among the preferred exercises implemented by sports practitioners, due to their high similarity to sport specific movements [8]. However, it has recently been shown that the correlations between muscle power outputs collected in the Olympic push press (OPP) at the OPL and sprinting and jumping performances were weaker (*r* = 0.40 to 0.48) than the correlations between JS power and these field-based performances (*r* = 0.71 to 0.86) [9]. Therefore, although correlations do not necessarily imply cause and effect, one could suspect that JS would produce greater gains in neuromuscular abilities than OPP in longitudinal interventions. Nevertheless, to date no study has addressed this question.

Therefore, the purpose of this study was to test and compare the changes in speed-power related abilities that occur in high-level under-20 soccer players after performing two different training regimens (i.e., JS or OPP exercises performed at the OPL) during a short-term preseason. To avoid misinterpretation of the data, both programs were identical in their settings (i.e., sets, intervals and repetitions).

## Methods

### Study Design

In this study a parallel two-group, matched, randomized, longitudinal design was conducted to test the effectiveness of JS or OPP exercises in high-level young soccer players, both performed at the OPL. During the study duration, which lasted 6 weeks into the preseason, apart from the JS or OPP training, both training groups were involved “solely” in different formats of small-sided games (SSGs) for technical-tactical development (Table 1). The SSGs were planned by the technical staff and consisted of 3 to 6 players per team, and small to medium pitch dimensions ranging from 12 × 20 m to 30 × 40 m, as put forth by Rampinini et al. [10]. Players were pair-matched according to their baseline performance in the 30m sprint test, and group allocation was performed by tossing a coin. All athletes had been previously familiarized with the coordinative patterns of the JS and OPP exercises and with the performance tests due to their professional testing and training routines. Besides assessing performance at the OPL of both exercises in the laboratory, the sprinting speeds at 5, 10, 20 and 30 m, change of direction (COD) speed and performance in the squat jump (SJ) and countermovement jump (CMJ) of the players were assessed as representative of field-based specific soccer physical components (pre- and post-training). Prior to all testing sessions, a general and specific warm-up routine was performed, involving light running (5-min at a self-selected pace followed by 3-min lower limb active stretching) and submaximal attempts at each testing exercise (e.g., submaximal sprints and vertical jumps). The warm-up prior to the JS or OPP training sessions comprised 5-min of jogging and smooth stretching exercises. In addition, players performed some submaximal movements with self-selected loads (2 x 8 with 2-min interval) with respect to the assigned exercise. Players attended the power training sessions in a non-fatigued state. During the experimental period, all soccer players performed 12 power-oriented training sessions, as follows: sessions 1–4) 6 x 8 JS or OPP using the optimum power load; sessions 5–8) 6 x 6 JS or OPP using 1.05 x the optimum power load; sessions 9–12) 6 x 4 JS or OPP using 1.10 x the optimum power load. A 2-minute interval was provided between each set of power-exercises. The JS training was performed in the Smith machine, whereas the OPP training was performed using an Olympic bar.

**Table 1:** Schematic representation of a typical weekly training schedule and total training volume during six weeks of a soccer preseason.

**Session**	**Monday**	**Tuesday**	**Wednesday**	**Thursday**	**Friday**		**Saturday**	
Morning	Power (JS or OPP)	SSG	Power (JS or OPP)	SSG	SSG			
Afternoon	SSG	Rest	SSG	SSG	Rest		FM	
**Total Weekly Volume (min/%)**	**1^st^ week**	**2^nd^ week**	**3^rd^ week**	**4^th^ week**	**5^th^ week**			**6^th^ week**
SSG	365’ (*73%)	310’ (54%)	330’ (61%)	400’ (65%)		420’ (61%)		380’ (74%)
Power (JS or OPP)	40’ (8%)	80’ (15%)	120’ (22%)	120’ (19%)		80’ (11%)		40’ (9%)
FM	90’ (19%)	180’ (31%)	90’ (17%)	90’ (16%)		180’ (28%)		90’ (17%)

Note: SSG = small-sided games training; Power training (jump squat or Olympic push press); FM = Friendly Match. SSG involved game formats with 3 to 6 players per team and small to medium pitch dimensions ranging from 12 × 20 m to 30 × 40 m.

(*) percentage of the total weekly training volume which this type of training represents.

### Subjects

Twenty-seven high-level U-20 male soccer players from the same 1^st^ division club (age: 18.4 ± 1.2 years, height: 1.78 ± 0.70 m, body mass: 74.4 ± 9.5 kg, training experience: 7.2 ± 1.9 years) took part in this investigation, after being informed of the potential benefits and risks associated with participation. The athletes were pair-matched in two training groups: jump squat group (JSG; n = 14) and Olympic push-press group (OPPG; n = 13). Ten players were excluded from the sample due to injuries unrelated to the proposed training/testing or transference to another team. Therefore, seventeen players completed the study (n = 9 and n = 8 for JSG and OPPG, respectively). The study protocol took place prior to the São Paulo State U-20 Soccer Championship, during the preseason training period. The study was approved by the Bandeirante Anhanguera University Ethics Committee conformed to the Declaration of Helsinki, and the participants and their legal guardians (in the case of <18 years of age) signed an informed consent form prior to research commencement.

### Vertical jumping tests

Vertical jumping height was determined using both SJ and CMJ. In the SJ, subjects were required to remain in a static position with a 90° knee flexion angle for 2-s before jumping. In the CMJ, the soccer players were instructed to execute a downward movement followed by a complete extension of the legs. The SJ and CMJ were executed with the hands fixed on the hips. All jumps were performed on a contact platform (Smart Jump; Fusion Sport, Coopers Plains, Australia). A total of five attempts were allowed for each jump, interspersed by 15-s. The best attempts at SJ and CMJ were retained.

### Bar mean propulsive power in jump squat and Olympic push-press

Bar mean propulsive power (MPP) in the JS and OPP exercises was assessed on a customized Smith machine (adapted by Hammer Strength, Rosemont, USA) and Multi-Hack equipment (Hammer Strength, Rosemont, USA), respectively. The soccer players were instructed to execute three repetitions at maximal velocity for each load, starting at 40% of their body mass (BM) in the JS and 30% of their BM in the OPP. In the JS exercise, athletes executed a knee flexion until the thigh was parallel to the ground (≈ 100° knee angle) and, after a command, jumped as fast as possible without losing contact between their shoulder and the bar. In the OPP exercise, athletes were required to start in the “rack position” (without countermovement) and accommodate the bar on the shoulders with the back of the arm parallel with the ground [11]. After a slight self-selected hip and knee flexion the athletes rapidly extended the hips and legs to generate upward momentum, and fully extended the arms to finish the movement with the bar overhead, in a full perpendicular trajectory (in relation to the ground). A load of 10% BM (in JS) and 5% BM (in OPP) was gradually added in each set until a decrease in mean propulsive power was observed. A 5-min interval was provided between sets. To determine MPP, a linear transducer (T-Force, Dynamic Measurement System; Ergotech Consulting S.L., Murcia, Spain) was attached to the Smith machine bar (in the JS) and the Olympic barbell (in the OPP). The respective starting loads and load progression allowed the determination of all individuals’ peaks of mean propulsive power consistently and accurately. The technical specification of the MPP analysis, its calculation and validity, and the respective validity of the equipment used to perform this measurement have been extensively reported in the literature [12–14]. The finite differentiation technique was used to calculate bar velocity and acceleration [14]. The bar maximum MPP values obtained were considered for data analysis purposes.

### Sprinting speed

Four pairs of photocells (Smart Speed, Fusion Equipment, AUS) were positioned at distances of 0, 5, 10, 20, and 30 m along the sprinting course. The soccer players sprinted twice, starting from a standing position 0.3 m behind the starting line. In order to avoid weather influences, the sprint tests were performed on an indoor running track. A 5-min rest interval was allowed between the two attempts and the fastest time was considered for the analyses.

### Zig-zag change of direction speed (Change of direction speed test)

**Fig 1:**
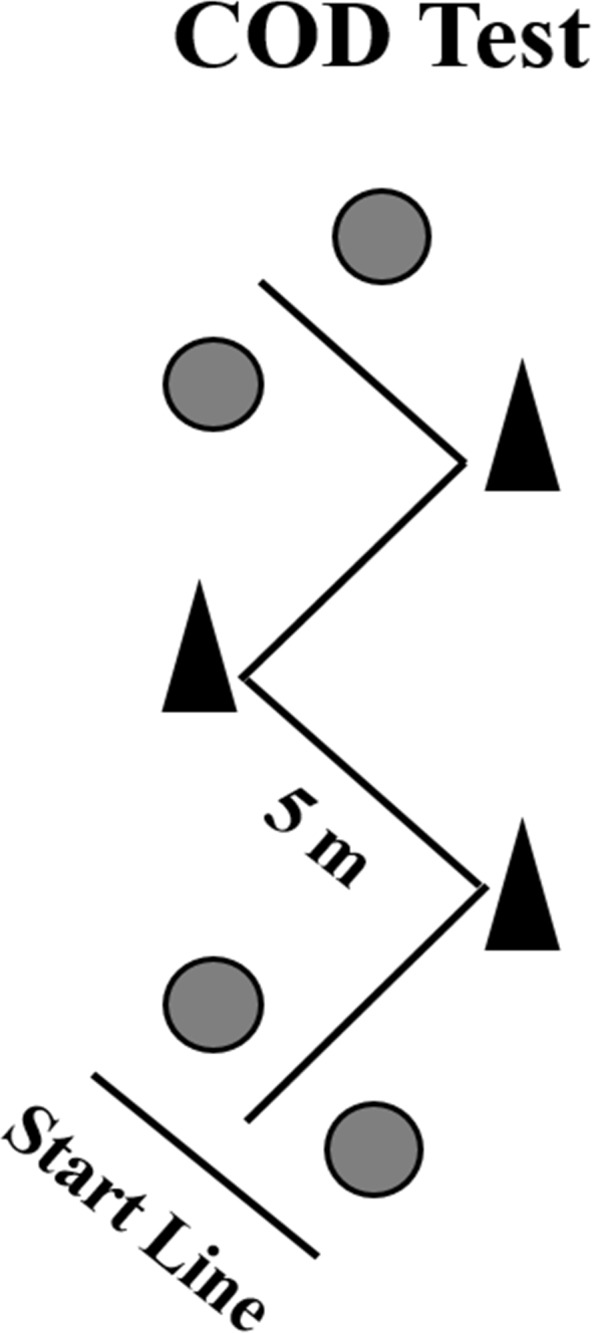
A schematic presentation of the Zig-zag COD speed test. The gray circles represent the position of the photocells.

The change of direction course consisted of four 5m sections marked with cones set at 100° angles, in an indoor court (Fig 1). The athletes were required to decelerate and accelerate as fast as possible without losing body stability. Two maximal attempts were performed with a 5-min rest interval between attempts. Starting from a standing position with the front foot placed 0.3 m behind the first pair of photocells (i.e., starting line), the athletes ran and changed direction as quickly as possible, until crossing the second pair of photocells, placed 20 m from the starting line [15]. The fastest time from the two attempts was retained for analyses.

### Statistical Analysis

Data are presented as mean ± standard deviation (SD). Both experimental groups were similar at baseline with respect to all dependent variables. To analyze the differences in the vertical jumps, sprinting velocity (VEL), COD speed, and MPP in the JS and OPP exercises in the JSG and OPPG pre and post-training, the differences based on magnitudes [16] were calculated. Moreover, the ANOVA two-way (group x time interaction) followed by the Bonferroni’s *post-hoc* (using IBM SPSS Statistics for Windows, Version 20.0. Armonk, NY: IBM Corp) was used to test for possible differences between groups. The quantitative chances of the JSG or OPPG, using a confidence interval of 90%, having higher, similar or lower values were assessed qualitatively as follows: <1%, almost certainly not; 1% to 5%, very unlikely; 5% to 25%, unlikely; 25% to 75%, possible; 75% to 95%, likely; 95% to 99%, very likely; >99%, almost certain. If the chances of having better and poorer results were both >5%, the true difference was assessed as unclear. The statistical level of significance was set as *P* < 0.05. Additionally, to determine the magnitude of the differences between the groups pre and post-training and its delta changes, the effect size (ES: Cohen’s d) was calculated [17]. The ES magnitudes were interpreted using the following thresholds [18]: <0.2, 0.2–0.6, 0.6–1.2, 1.2–2.0, 2.0–4.0, and >4.0 for trivial, small, moderate, large, very large, and near perfect, respectively.

To calculate the transfer effect coefficient (TEC) between MPP in the JS and OPP exercises, and the 5, 10, 20, and 30 m sprinting performance, we used the equation proposed by Zatsiorsky and Kraemer [19] as follows:

$$TEC=\frac{result\;gain\;in\;untrained\;exercise}{result\;gain\;in\;trained\;exercise}$$

TECs were only calculated for variables presenting an ES of at least 0.2, which is considered a small effect size [18].

Coefficients of variation (CV) and intraclass correlation coefficients (ICCs) were used to indicate the absolute and relative reliability, respectively, for OPP and JS exercises (for MPP), SJ and CMJ (for vertical jumping height), and sprinting and COD tests (for mean VEL). The ICC and CV were 0.94 and 3.8% for OPP; 0.96 and 4.1% for JS; 0.96 and 3.1% for SJ; 0.94 and 3.5% for CMJ; 0.97 and 2.3% for 30-m speed; and 0.96 and 2.4% for the COD test.

## Results

Both groups were similar at PRE for all assessed variables. Table 2 presents the vertical jumping performance and MPP in the JS and OPP exercises, at the pre- and post-training moments, in both the JSG and OPPG, along with the between group differences in the changes. After training, the vertical jump heights (SJ and CMJ) and the MPP in the JS exercise presented *likely* to *almost certain* improvements in the JSG (ES = 0.53, 0.38 and 1.30, respectively), while the OPPG presented an *almost certain* improvement only in the OPP exercise (ES = 1.23). The JSG presented higher improvements in the SJ, CMJ, and MPP JS than the OPPG (ES ranging from 1.32 to 3.18), while the OPPG presented greater improvement in the MPP OPP than the JSG (ES = 2.49) (interaction effect [group x time], *P* < 0.05) (Table 2). Additionally, pooling pre- and post- values, the OPP exercise presented *likely* (00/16/84) higher MPP values in comparison to the JS exercise (712.92 ± 113.34 W vs. 755.74 ± 145.95 W; for JS vs. OPP exercises; ES = 0.33).

**Table 2:** Vertical jumps and mean propulsive power (MPP) in the jump squat (JS) and Olympic push press (OPP), pre and post 6 weeks of preseason in under-20 soccer players.

	Group	JSG	OPPG	Between group differences in the change *Rating*
	Pre	38.31 ± 4.26	36.39 ± 4.87	ES: 1.42 (0.43; 2.19) *Large*
SJ (cm)	Post	40.56 ± 3.78	35.86 ± 3.97	98/02/00 *Very Likely*
	Δ%	5.9 (3.6–8.2)	-1.5 (-5.1–2.2)*	
	Pre	40.07 ± 4.74	37.15 ± 4.67	ES: 1.32 (0.32; 2.05) *Large*
CMJ (cm)	Post	41.86 ± 4.42	37.51 ± 4.11	85/15/00 *Likely*
	Δ%	4.4 (3.5–5.4)	0.9 (-1.0–2.9)*	
	Pre	688.00 ± 123.88	688.22 ± 98.02	ES: 3.18 (1.50; 3.66) *Very large*
MPP JS (W)	Post	849.25 ± 173.63	714.16 ± 97.59	100/00/00 *Almost Certainly*
	Δ%	23.4 (15.3–31.6)	3.8 (2.6–4.9)*	
	Pre	725.50 ± 172.98	721.33 ± 92.94	ES: 2.49 (1.27; 3.33) *Very large*
MPP OPP (W)	Post	760.60 ± 182.54	826.98 ± 107.83	00/01/99 *Very Likely*
	Δ%	4.8 (2.6–7.1)	14.7 (11.6–17.8)*	

Note: Δ%: percentage of change (90% confidence limits); ES: effect size (90% confidence limits); SJ: squat jump; CMJ: countermovement jump; JSG: jump squat group; OPPG: Olympic push press group.

*Interaction effect (group x time), P < 0.05.

Table 3 presents the VEL 5, 10, 20 and 30 m and COD speed pre- and post-training comparisons, along with the between group differences in the changes. The sprinting velocity in all distances (5, 10, 20, and 30 m) and the COD speed presented *almost certain* improvements after training in the JSG (ES = 1.68, 1.45, 1.26, 0.94, 1.00, and 0.94, respectively), while in the OPPG the sprinting and COD performances did not change substantially (ES = 0.23, 0.06, 0.01, -0.10, and 0.09, for VEL 5, 10, 20 and 30 m, and COD, respectively). Additionally, the changes in the sprinting and COD performances were *very likely* to *almost certainly* higher in the JSG in comparison to the OPPG (ES ranging from 1.26 to 2.64) (interaction effect [group x time], *P* < 0.05) (Table 3).

**Table 3:** Sprinting velocity (VEL) and change of direction (COD) speed, pre and post 6 weeks of preseason in under-20 soccer players.

	Group	JSG	OPPG	Between group differences in the change
				*Rating*
	Pre	4.95 ± 0.22	5.16 ± 0.30	ES: 1.47 (0.51; 2.30) *Large*
VEL 5 m (m.s^-1^)	Post	5.32 ± 0.16	5.23 ± 0.27	99/01/00 *Very Likely*
	Δ%	7.7 (4.7–10.5)	1.4 (-1.2–3.9)*	
	Pre	5.83 ± 0.22	5.92 ± 0.34	ES: 1.92 (0.87; 2.78) *Large*
VEL 10 m (m.s^-1^)	Post	6.15 ± 0.17	5.94 ± 0.26	100/00/00 *Almost Certainly*
	Δ%	5.5 (3.8–7.0)	0.3 (-1.4–2.0)*	
	Pre	6.77 ± 0.19	6.90 ± 0.35	1.74 (0.69; 2.53) *Large*
VEL 20 m (m.s^-1^)	Post	7.01 ± 0.17	6.90 ± 0.30	99/01/00 *Very Likely*
	Δ%	3.6 (2.5–4.6)	0.0 (-1.5–1.5)*	
	Pre	7.33 ± 0.22	7.42 ± 0.39	ES: 2.64 (1.44; 3.57) *Very large*
VEL 30 m (m.s^-1^)	Post	7.55 ± 0.21	7.38 ± 0.35	100/00/00 *Almost Certainly*
	Δ%	3.0 (2.1–3.8)	-0.7 (-1.5–0.3)*	
	Pre	3.60 ± 0.16	3.63 ± 0.11	ES: 1.65 (0.64; 2.46) *Large*
COD Speed (m.s^-1^)	Post	3.75 ± 0.15	3.64 ± 0.14	99/01/00 *Very Likely*
	Δ%	4.2 (2.8–5.6)	0.3 (-1.4–1.9)*	

Note: Δ%: percentage of change (90% confidence limits); ES: effect size (90% confidence limits); JSG: jump squat group; OPPG: Olympic push press group.

*Interaction effect (group x time), P < 0.05.

TEC was calculated between the trained (JS and OPP) and untrained (5, 10, 20, and 30 m sprinting VEL) exercises. The TEC between the JS and VEL 5, 10, 20, and 30 m were 1.29, 1.12, 0.97, and 0.77, respectively. The only TEC that could be calculated in the OPPG (between OPP and VEL 5 m [TEC = 0.20]) was rather low.

## Discussion

This study aimed to compare the neuromuscular adaptations provided by two different complex multi-joint exercises (i.e., JS or OPP) performed at the optimum power zone. Although both exercises were *almost certainly* effective for specifically increasing their respective muscle power capacities (i.e., MPP JS and MPP OPP), in accordance with our expectations, the JS was more effective for developing speed-power related abilities in high-level young soccer players. This apparent superiority is probably related to the neuromechanical characteristics of these ballistic exercises.

Concerning this issue, it has already been reported that the OPP is able to produce higher power outputs than the JS, besides avoiding the “impact forces” (i.e., ground reaction forces) that arise from the landing phase [9,11]. Indeed, our results confirmed this observation and the athletes were capable of generating greater values of MPP in the OPP in comparison to the JS. From a mechanical perspective, it is highly predictable that the concomitant use of lower and upper extremities throughout the OPP execution would be capable of optimizing its muscle power production [11,20]. Nevertheless, it seems that this “boosting effect” does not positively affect the training outcomes, at least for the specific development of speed and vertical jumping abilities. Possibly, the increased mechanical impulse (at the ankle, knee and hip joints) provided by the simultaneous extension of lower and upper limbs creates an unusual neuromechanical condition, very different from that found in sprinting and jumping activities [21].

Indeed, it is well established that the hip and knee muscles play a crucial and central role in speed and jump performances [22,23]. Importantly, the relative contribution of the hip muscles to the total torque produced in the lower extremities during sprinting and vertical jumping activities tends to increase with increasing intensity [24,25]. In the same way, in lower limb movements, the hip joint extension moment increases as loading intensity increases [23]. Thus, it seems that the maximal development of the hip extension ability is directly dependent on the “training-loading strategy”. In this sense, it is worth highlighting the mechanical differences in the *force-momentum* balance during execution of both the OPP and JS. In physics, the *momentum* is defined as the product of mass and its relative velocity (*i*. *e*., *momentum* = *mass* × *velocity*) [26]. Throughout the OPP exercise, the synchronized use of arms and legs enables athletes to achieve very high bar-velocities (using very-light loads), thus compromising the mechanical balance of the *momentum* equation. Therefore, despite the increased mechanical outputs in this complex “full-body movement”, the load intensity seems to be significantly compromised in OPP. Conversely, the JS OPL occurs at a more balanced mechanical zone, with the subjects moving moderate (to heavy) loads at moderate (to high) velocities. In addition, during the JS, the absence of the inertial component caused by the concomitant action of the arms (which occurs during OPP) possibly results in a greater recruitment of hip and knee joint muscles. Undoubtedly, this neuromechanical sequential pattern is much more related to speed and jumping abilities than the unusual concomitant extension of arms and legs.

In fact, the ballistic JS has been extensively correlated to jumping and sprinting performance [27–29]. Partly, this association may be clarified when analyzing the kinematics and kinetics characteristics of this explosive exercise. For jumping higher (and consequently producing higher muscle power outputs), an athlete has to apply great amounts of force against the ground, as rapidly as possible [30]. Essentially, the performance in JS is highly dependent on the athlete’s ability to execute fast and forceful concentric actions, by simultaneously using the knee and the hip extensor muscle groups [31,32]. Likewise, in sprinting performance, the hip and knee muscle joints can be considered as the most important contributors to produce the highest levels of speed in elite athletes [33]. Furthermore, throughout the JS executions the athletes achieve superior ranges of motion than throughout the OPP executions, which possibly leads to different magnitudes of neuromuscular adaptations. It is well established that the “deeper movements” (e.g., higher levels of hip and knee flexion) elicit favorable adaptations on knee extensors [34], a muscle group which play a determinant role in sprint and jump performance. Possibly, these mechanical similarities positively affected the speed ability in the JS group, thus increasing the transference effect of JS outputs on sprinting performance.

The transference calculation is a statistical tool designed to estimate the transfer effect of the gains in the *“trained exercise”* on the *“untrained exercise”* [7,19]. In this study, besides the experimental interventions (i.e., JS and OPP exercises), the young soccer players solely performed small-sided games (SSG). Since it is well established that SSGs are unable to improve speed ability [7,35–37], it is reasonable to consider that both the JS and OPP exercises could have caused the changes in sprinting performance after the experimental intervention. This assertion can be easily confirmed when analyzing the distinct changes in sprinting speed presented by the groups at the end of the training period. The higher effect sizes observed in the JS group for all tested distances (VEL 5, 10, 20, and 30 m) could produce greater values of *transfer effect* in this group [19]. On the other hand, in the OPP group, the absence of, as minimum, small increases in speed ability precluded the calculation of the transference between trained (OPP) and untrained (sprint) exercises.

Curiously, the COD speed performance was only improved in the JS group. Although COD ability seems to depend on multiple factors [38,39], it is possible that the JS mechanics equally favors this capacity. Indeed, Hewit et al. [38] identified the “key technical features” of COD performance, highlighting the importance of rapidly applying high levels of force against the ground at the takeoff, which enables athletes to accelerate in the new direction as quickly as possible. This rapid “concentric force development” is also critical for generating greater levels of muscle power during the vertical jumps [40,41]. Nevertheless, despite the important increases in MPP JS, the JS group presented only small improvements in the vertical jumping ability (i.e., assessed by means of SJ and CMJ height). These findings are possibly related to the distinct mechanisms involved in jumping activities performed under loaded or unloaded conditions. Due to the external overloading, the loaded JS is executed at lower velocities than the SJ and CMJ. Thus, to some extent, the performance in JS is dependent on the ability to produce greater amounts of force at lower speeds (i.e., maximum dynamic strength) [42,43]. Conversely, the performance in unloaded vertical jumps (i.e., SJ and CMJ) seems to be more dependent on precise and specific adjustments in the contractile machinery (i.e., neuromuscular structures responsible for jumping pattern coordination) [44,45]. Conceptually, to enhance these particular neuromechanical capacities the soccer players should have included a substantial number of specific plyometric sessions (e.g., drop jump sets) in their training routines [7,40,46]. From these findings, it should be considered the necessity of adopting mixed training models (i.e., strength-power training combined with plyometrics) in order to achieve higher and more consistent neuromuscular adaptations in top-level soccer players.

Of note, the range of loads used in this study was very effective for eliciting positive (and larger) adaptations in MPP in both the JS and OPP exercises. As aforementioned, it has been demonstrated that the OPL is capable of developing the strength-power abilities at both ends of the force-velocity curve [5,47], apart from being closely associated with sport specific performance [28,29,48–50]. However, surprisingly, even with the expressive gains presented by the OPP group in the MPP OPP, the subjects were unable to transfer these “positive adaptations” to sprint and jump performance. From a practical standpoint, it seems that the “correct transference” of muscle power to speed-power related abilities depends not only on the training intensity, but rather, on the appropriate choice of exercise techniques. In this regard, at least for the physical abilities directly related to the capacity to rapidly apply higher levels of force against the ground (i.e., short-sprint ability), the ballistic loaded JS should be preferred over the multi-complex OPP exercise. Possibly, these outcomes are directly connected to the mechanical characteristics of these exercises, including their execution *per se* and the distinct ranges of motion achieved in each exercise. This novel finding may have important implications on strength-power training prescription.

We recognize that the absence of a control group (e.g., a sprint training group) is a limitation of this investigation. However, this study was performed with top-level soccer players during a short-term preseason (before an important competitive season), which strongly restricts the experimental possibilities.

## Conclusion

The findings of this study support adopting the JS exercise to the detriment of the OPP in soccer players, due to its superior transference effects on sprinting over short (5 m) and long (30 m) distances. The use of JS could be a safe strategy for increasing speed ability in soccer players, without the inherent risks involved in maximal sprint training (e.g., hamstring injuries) [51]. Possibly, this superiority is related to the mechanical characteristics of the JS, which closely resemble the “segmental triple extension” elicited during the sprinting strides. Importantly, the JS is a safe exercise and does not require the same level of coordination demanded in Olympic weightlifting [52]. For these reasons, it can be easily implemented in soccer training routines and should be preferred in comparison to the more complex OPP. Further studies should be conducted to test the effectiveness of combining JS with plyometrics to improve the plethora of field-related physical capacities demanded by soccer.
